# COVID-19 Vaccine-Related Myocarditis: A Descriptive Study of 40 Case Reports

**DOI:** 10.7759/cureus.21740

**Published:** 2022-01-30

**Authors:** Jia Hong Chen, Ifeanyi A Ikwuanusi, Veera Jayasree Latha Bommu, Vraj Patel, Harpreet Aujla, Vishrut Kaushik, Pramil Cheriyath

**Affiliations:** 1 Internal Medicine, Hackensack Meridian Ocean Medical Center, Brick, USA; 2 Medicine, American University of Antigua, Antigua, USA

**Keywords:** covid-19 vaccine related adverse events, pericarditis, myocarditis, vaccine related myocarditis, janssen, and jnj-78436735 (johnson & johnson), mrna-1273 (moderna), bnt162b2 (pfizer-biontech), covid-19 vaccine

## Abstract

After the surging rise in the Coronavirus disease 2019 (COVID-19) pandemic, the Food and Drug Administration (FDA) approved emergency approval of vaccinations to prevent life-threatening complications of COVID-19 infection. These vaccines are BNT162b2, mRNA-1273. Later, the FDA also approved JNJ-78436735. COVID-19 vaccination does not have major side effects, but there are some concerning adverse events reported right after vaccination. Myocarditis is one of them. Based on our analysis of 40 case reports, we are presenting the epidemiology and clinical picture of myocarditis related to the COVID-19 vaccine.

Based on our analysis, we found that the majority of cases were seen in males with 90% predominance, and these cases were seen in the age group of 29.13 years old (mean, SD of 14.39 years). In 65% of cases, patients took the BNT162b2 vaccine; 30% of cases were reported with the mRNA-1273 vaccine; and 5% of cases with JNJ-78436735. Of all the cases, 80% of them are reported after the second dose of the vaccine with either Moderna or Pfizer.

The characteristics of COVID-19 vaccine-related myocarditis were analyzed in this study. We identified several findings, ranging from age, gender, type of vaccination, presentation of symptoms, and diagnosis modality. This depicts the picture of COVID-19 vaccine-related myocarditis and what physicians should expect when dealing with the disease. Our analysis showed that more cases were reported after receiving the BNT162b2 vaccine compared to mRNA-1273 and JNJ-78436735 vaccines. Further research needs to be conducted to analyze the underlying cause of this association.

## Introduction and background

The US Food and Drug Administration (FDA) approved two mRNA vaccines and a Janssen vaccine that were designed to prevent Coronavirus disease 2019 (COVID-19)-related lethal complications and mortality [1]. As of January 2021, 9.21 billion doses of COVID-19 vaccines have been administered globally [2]. In a prospective observational study by Menni et al., 159,101 (25.4%) of 627,383 reported one or more systemic adverse effects, while 257,209 (66.2%) of 388,430 reported one or more local adverse effects after the BNT162b2 vaccine [3]. As of December 16, 2021, the United States Centers for Disease Control and Prevention (CDC) VAERS (Vaccine Adverse Event Reporting System) has received 1,947 preliminary reports of myocarditis or pericarditis among patients aged 30 and younger who have received COVID-19 vaccinations. Myocarditis/pericarditis rates among people aged 12 to 39 are 12.6 cases per million doses of second-dose mRNA vaccine, according to the US Centers for Disease Control and Prevention [4]. The objective of this study is to understand the epidemiological and clinical picture of COVID-19 vaccine-related myocarditis.

## Review

Methods

Eligibility Criteria 

We included case reports and case series that developed myocarditis following COVID-19 vaccination, regardless of the type of vaccination, that were published online and excluded all other COVID-19 vaccine-related adverse events. We found around 25 studies that met our criteria. After excluding duplication, we finally included 14 published articles in our study, which consists of 40 case reports (Table 1) [1,5-17] of myocarditis after receiving the COVID-19 vaccine.

**Table 1:** References of the case reports and case series used in this study

No	Authors	Study name	Brief description
1.	Nassar et al. [1]	COVID-19 vaccine-induced myocarditis: a case report with literature review	Myocarditis is a likely consequence of COVID-19 vaccinations, according to the results of this case scenario. To avoid fatal complications, a differential assessment of patients with COVID-19 vaccination status and signs of severe cardiac decompensation must rule out myocarditis.
2	Lim et al. [5]	Case report: acute fulminant myocarditis and cardiogenic shock after messenger RNA Coronavirus disease 2019 vaccination requiring extracorporeal cardiopulmonary resuscitation	After mRNA COVID-19 vaccination, a case of acute fulminant myocarditis was worsened by cardiogenic shock, and the efficiency of ECMO assistance was critical for life-saving.
3	Onderko et al. [6]	Myocarditis in the setting of recent COVID-19 vaccination	Three patients developed chest pain after receiving the BNT162b2 Pfizer/BioNTech or the mRNA-1273 Moderna vaccine. Myocarditis was confirmed by clinical signs, biomarkers, and cardiac MRI.
4	Tailor et al. [7]	Case report: acute myocarditis following the second dose of mRNA-1273 SARS-CoV-2 vaccine	After receiving the second dosage of the mRNA-1273 SARS-CoV-2 vaccine, a patient developed symptomatic acute myocarditis. Minimal coronary artery disease was discovered during an emergency coronary angiography. Acute myocarditis was verified by cardiac magnetic resonance imaging (cMRI).
5	Albert et al. [8]	Myocarditis following COVID-19 vaccination	After receiving COVID-19 immunization, a 24-year-old had myocarditis symptoms. Myocarditis is a rare side effect of the COVID-19 vaccination, according to a case study.
6	Gautam et al. [9]	A late presentation of COVID-19 vaccine-induced myocarditis	A 66-year-old man presented with acute-onset chest pain after three months of receiving the second dose of the mRNA vaccine. On cMRI, he was discovered to have acute myocarditis, which was related to his exposure to the COVID-19 vaccine in the absence of any other risk factors. Review of the literature and summarized published case reports on COVID-19 vaccine-associated myocarditis.
7	Marshall et al. [10]	Symptomatic acute myocarditis in seven adolescents after Pfizer-BioNTech COVID-19 vaccination	This is a case series of seven cases of acute myocarditis or perimyocarditis in healthy male teenagers who developed chest pain four days after receiving the second dose of the Pfizer-BioNTech COVID-19 vaccine. Troponin levels were abnormally high in all of the patients. Late gadolinium enhancement (LGE) was seen on cardiac MRI, which is a sign of myocarditis. The symptoms of all seven patients disappeared quickly. Three patients received only nonsteroidal anti-inflammatory medications, whereas four others received intravenous immunoglobulin as well as corticosteroids.
8	Starekova et al. [11]	Myocarditis associated with mRNA COVID-19 vaccination	Five patients with aberrant MRI findings (4:1 male/female; 17–38 years old) were identified and were vaccinated against COVID-19 prior to MRI. All of the patients' cardiac troponin levels and ECG findings were abnormal. All of the patients were admitted to the hospital after experiencing sudden chest pain and being diagnosed with acute myocarditis.
9	Salah and Mehta [12]	COVID-19 vaccine and myocarditis	This pooled analysis showed that COVID-19 vaccine-related myocarditis mostly occurs in young male individuals following the second dose of the vaccine. It also showed that most cases occurred with mRNA vaccines (i.e., Pfizer-BioNTech and Moderna). And in all the reported cases, clinical symptoms resolved within six days with preservation of the cardiac function.
10	Hasnie et al. [13]	Perimyocarditis following the first dose of the mRNA-1273 SARS-CoV-2 (Moderna) vaccine in a healthy young male: a case report	A case of perimyocarditis that was temporally related to COVID-19 mRNA vaccination after three days of vaccination in a young male with prior COVID-19 infection but otherwise healthy.
11	Nagasaka et al. [14]	Acute myocarditis associated with COVID-19 vaccination: a case report	A case of myocarditis three days following a Pfizer COVID-19 vaccine.
12	Deb et al. [15]	Acute myocardial injury following COVID-19 vaccination: a case report and review of current evidence from vaccine adverse events reporting system database	A 67-year-old man presented with acute congestive heart failure and sustained acute myocardial damage as a result of an acute immunological response following the second dose of COVID-19 immunization (Moderna). Over the course of three days, his clinical condition improved.
13	Singh et al. [16]	COVID-19 mRNA vaccine and myocarditis	Case report of a 24-year-old male patient who developed chest pain after receiving the second dose of the Pfizer-BioNTech COVID-19 vaccine. He was diagnosed with myocarditis on further workup.
14	Tinoco et al. [17]	Perimyocarditis following COVID-19 vaccination	A case of a 39-year-old healthy individual having chest pain diagnosed with myocarditis three days after receiving the second dose of COVID-19 vaccine.

Search Strategies

A systematic search of COVID-19 vaccine-related myocarditis case reports utilizing Google and scientific databases such as Google Scholar and PubMed.

Data Collection Process and Data Items

Using standardized data extraction forms, the data were extracted independently by two authors. We collected characteristics like age, gender, and other variables like the type of vaccination, sign/symptoms, how many days after they were diagnosed with myocarditis, outcome, laboratory values, methods of diagnosis, and results on an Excel sheet (Microsoft Corporation, Redmond, WA), and these variables were analyzed.

Statistical Analysis

Patient demographic characteristics, disease manifestations, and causes were summarized descriptively and analyzed using R version 1.1.456 (RStudio: Integrated Development for R, RStudio, PBC, and Boston, MA).

Results

**Figure 1:**
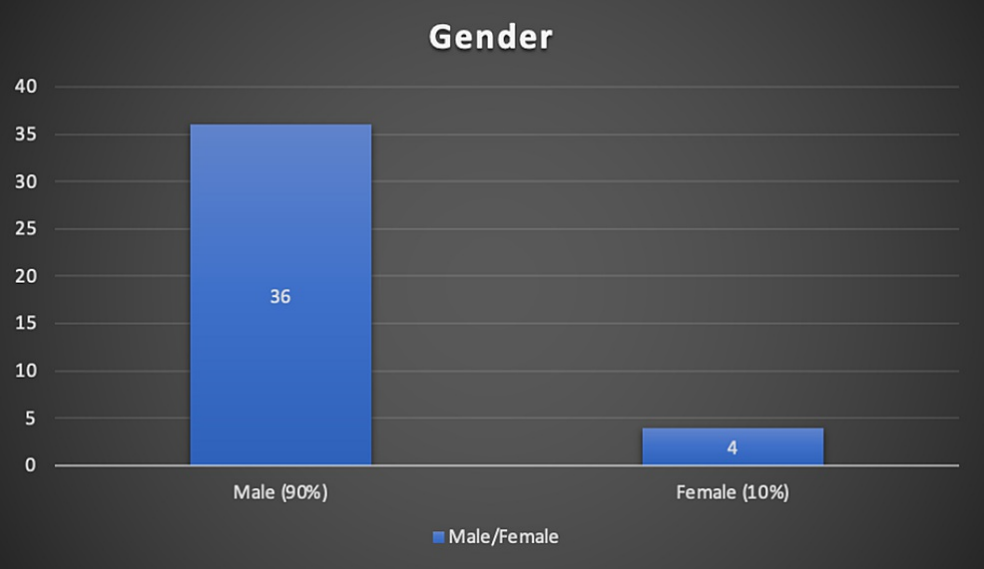
Percentage of males versus females affected

In this study of 41 case reports of COVID-19 vaccine-induced myocarditis, the average age of presentation was 29.13 ± 14.39 years of age. Out of all reported cases, 90% of cases are seen in males and only 10% of cases are seen in females (Figure 1).

**Figure 2:**
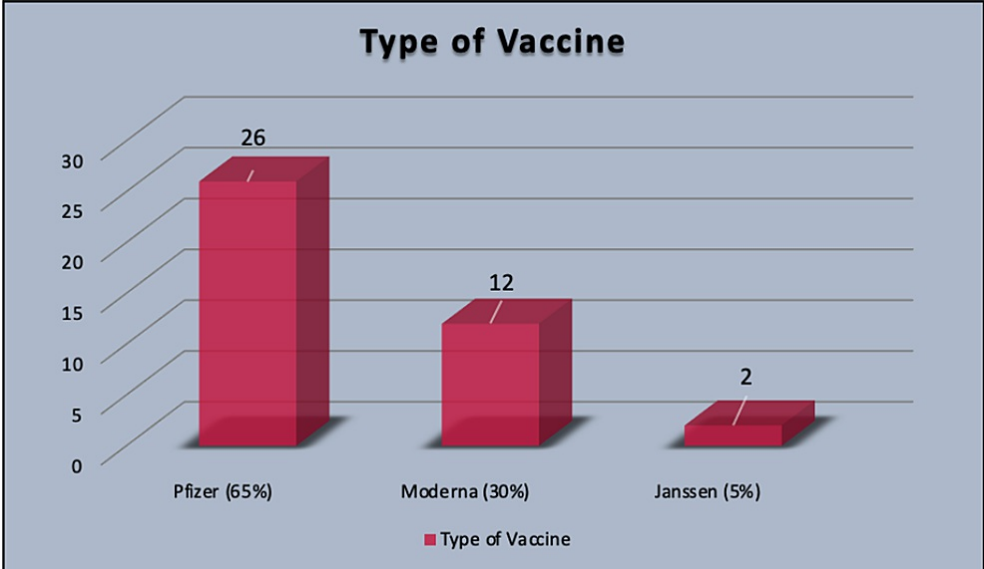
Percentage of myocarditis with various types of vaccine

The majority of the reported cases got myocarditis after taking the Pfizer vaccine, which in 26 cases was around 65%, while 12 patients took Moderna at 30%. Out of these 40 reported cases, only 2 (5%) took the Janssen (J&J) vaccine (Figure 2).

**Figure 3:**
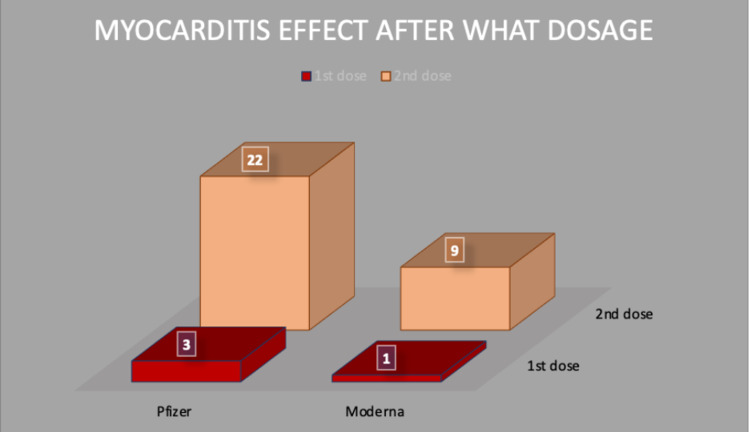
Number of myocarditis cases after each dosage

In the collected data, we found most of the cases, 32, were after the second dose of vaccine (80%), three cases were reported after the first dose of vaccine (7.50%), and five cases were not reported anything specific to the dose (13%) for Pfizer and Moderna vaccines (Figure 3).

We found that almost all patients' data had normal vitals, with an average systolic blood pressure of 119.72 mmHg with a ±22.36 mmHg range, while diastolic blood pressure was 71.44 mmHg with ±10.65 mmHg range. The overall heart rate was measured at 93.17 ± 21.41 beats per minute. SpO_2_ saturation was noted at 94% ± 7.9% (Table 2).

**Table 2:** Various parameters in patients with COVID-19 vaccine-induced myocarditis EKG: electrocardiogram; CRP: C-reactive protein; BNP: brain natriuretic peptide

Parameters	Values (±standard deviation SD)
Age	29.125 (±14.39) years
Gender	36 males (90%); 4 females (10%)
Vitals	
Systolic blood pressure	119. 72 (±22.36) mmHg
Diastolic blood Pressure	71.44 (±10.65) mmHg
Heart rate	93.17 (±21.41) beats per minute
SpO_2_	94% (±7.9%)
Type of vaccination	Pfizer - 26 (65%); Moderna - 12 (30%); Janssen (J&J) - 2 (5%)
After which dose of vaccine they developed symptoms	1^st^ dose: 3 cases (7.50%) 2^nd^ dose: 32 cases (80%) Unknown: 5 cases (13%)
How many days after vaccination patient hospitalized?	5.09 days (±14.04 days)
EKG changes	Number of cases (percentage)
ST-elevation	25 (62.50%)
Normal	8 (20%)
T-wave changes	5 (13%)
PR depression	2 (5%)
Laboratory values	Values (±standard deviation)
Peak troponin I level	162.275 ng/mL (±754.804 ng/mL)
CRP	26.43 mg/L (±31.98 mg/L)
BNP	51.31 pg/ml (±25.64 pg/ml)

**Figure 4:**
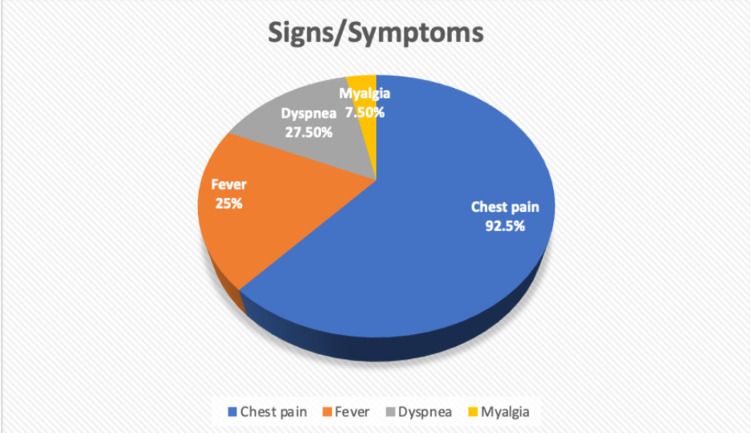
Clinical features of COVID-19 vaccine-related myocarditis

The majority of the patients were noted to have chest pain (92.5%), with 27.50% having common complaints with dyspnea. 25% of patients had associated fever and 7.50% had myalgia (Figure 4).

Most of the patients who presented with chest pain were later diagnosed with myocarditis, majority of the patients had electrocardiogram (EKG) changes, with most cases, 25 reported cases (62.50%) were seen with ST elevation in various leads, five cases reported T-wave changes (13%), and two cases mentioned PR depression (5%). Overall peak troponin-I level was 162.275 ng/ml with a standard deviation of ±754.804 ng/mL. C-reactive protein (CRP) level was 26.43 mg/L with a SD of ±31.98 mg/L and the brain natriuretic peptide (BNP) was 51.31 pg/ml ± 25.64 pg/ml (Table 1).

COVID-19 PCR tests were performed in all reported cases, and all patients tested negative for COVID-19 infection. Reported ejection fraction in echocardiography comes to an average of 51% with a standard deviation of 10.83%. Following echocardiography and EKG, only 22 patients underwent coronary angiography. Of those, 20 cases had no luminal irregularities, one case had non-obstructive coronary artery disease in the first septal perforator branch, and the other case had minimal coronary artery disease. Most patients were diagnosed with myocarditis after undergoing cardiac MRI, which showed cardiac wall edema on gadolinium enhancement with signs of fibrosis or hyperemia. Out of 40 cases, 32 cases (80%) reported myocardial wall edema on gadolinium enhancement in the case reports.

Most of the reported cases in the analysis recovered from myocarditis. 39 (97.5%) cases recovered from myocarditis and were discharged from the hospital, where one case (2.5%) concluded in the demise of the patient. The 2.5% of mortality is concerning and needs further research studies on COVID-19 vaccines causing myocarditis to explore more. The average discharge from the hospital was around 6.375 days after hospitalization, with a standard deviation of ±11.61 days. The median value for discharge was four days, and a maximum number of patients got discharged after two days of hospitalization after improvement in the symptoms.

Discussion

Since the World Health Organization (WHO) declared COVID-19 a pandemic on March 11, 2020, it has affected tens of millions of people worldwide. To contain the pandemic, which has had severe medical, economic, and social implications, safe and efficient prophylactic vaccinations were urgently needed [18]. The discovery and approval of vaccines for the prevention of COVID-19 infection via Emergency Use Authorization is a big step forward in controlling the disease and lowering morbidity and mortality [19].

COVID-19 mRNA vaccines contain nucleoside-modified mRNA that encodes the SARS-CoV-2 viral spike glycoprotein and is encapsulated in lipid nanoparticles, but no live virus or DNA. Once created in the cell after the mRNA-vaccine entrance, the viral spike protein triggers an adaptive immune response to recognize and eliminate viruses that express the spike protein. Vaccine-induced spike-protein IgG antibodies neutralize SARS-CoV-2 by preventing it from attaching to the host cell (which occurs via spike-protein binding to the angiotensin-converting enzyme 2 receptors) [20].

Pain at the injection site, swelling, and redness were the most commonly reported local adverse effects of the COVID-19 vaccine. Fever, myalgia, tiredness, and headaches were among the systemic responses. Laboratory abnormalities such as reduced hemoglobin, increased bilirubin, and altered serum aspartate aminotransferase (AST) and alanine aminotransferase (ALT) were also noted in several trials. None of these changes were clinically noticeable and were self-contained [21].

Myocarditis is an inflammation of the heart's middle layer that leads to myocardial damage but no ischemic events [1]. The SARS-CoV-2 virus itself can cause myocarditis. The cytokine storm syndrome can exacerbate the pathogenesis of viral myocarditis, which is a combination of direct cell damage and T-lymphocyte-mediated cytotoxicity. The major mediator of cytokine storm appears to be interleukin 6 (IL-6), which orchestrates proinflammatory responses from immune cells, particularly T-lymphocytes [22].

Molecular mimicry between the SARS-CoV-2 spike protein and self-antigens is another major possible cause of myocarditis. In the laboratory, antibodies against SARS-CoV-2 spike glycoproteins have been demonstrated to cross-react with structurally comparable human peptide-protein sequences, including myosin [23]. In general, myocarditis is regarded as a rare side effect of immunization. Due to the low risk of myocarditis and the small number of people received the vaccination before it was approved, it is likely that uncommon adverse responses to the COVID-19 vaccine were missed during clinical trials. Vaccinating a large number of people in a short amount of time may enable the detection of less common vaccination-related events [19]. Clinical trials using COVID-19 vaccines were underpowered to detect the few adverse events that are important for risk-benefit evaluations and for influencing post-vaccination clinical treatment. As a result, finding such unusual negative outcomes has become a top scientific goal around the world [24].

The hypothesis of pathophysiology of vaccine-related myocarditis is known to be due to the immune system's mistaking the vaccine's mRNA for an antigen, triggering pro-inflammatory cascades and immunological pathways in the heart. Although nucleoside alterations lower mRNA's innate immunogenicity, the immune response to mRNA may still trigger an abnormal innate and acquired immune response, which could explain why mRNA vaccines elicit a higher immune response than other types of COVID-19 vaccination. Antibodies designed against SARS-CoV-2 spike glycoproteins may react with human protein sequences that are structurally similar, such as cardiac-myosin heavy chains [20]. This explains the higher number of cases following mRNA vaccines (Pfizer and Moderna) [95%] compared to Janssen vaccines [5%]. Another hypothesis is that having an autoimmune disease is an independent risk factor for adverse events such as myocarditis, atrial fibrillation, and rhabdomyolysis after receiving COVID-19 vaccines. When activated by the vaccine, a dysregulated immune system in autoimmune diseases may begin to produce autoantibodies against cardiac myocytes or cardiac ion channels [25].

Our analysis showed that more cases of myocarditis were reported in our analysis among people who received the Pfizer vaccine than among people who received the Moderna vaccine. Our hypothesis is that more people are receiving Pfizer vaccines compared to Moderna. This might be the reason for the higher number of reported cases of myocarditis with Pfizer. Thus, further research needs to be conducted for the detailed evaluation of the underlying reason for the same, although both the vaccines are mRNA vaccines and have an almost similar composition (Table 3) [26-28].

**Table 3:** Components of Pfizer versus Moderna vaccine versus Janssen References [26-28]

Pfizer vaccine (BioNTech)	Moderna vaccine (mRNA-1273)	Janssen (JNJ-78436735)
Messenger ribonucleic acid (mRNA)	Messenger ribonucleic acid (mRNA)	Recombinant, replication-incompetent adenovirus type 26 expressing the SARS-CoV-2 spike protein
Lipids (their main role is to protect the mRNA and provide somewhat of a “greasy” exterior that helps the mRNA slide inside the cells). ((4-hydroxybutyl)azanediyl)bis(hexane-6,1-diyl)bis(2-hexyldecanoate), 2 [(polyethylene glycol)-2000]-N, N-ditetradecylacetamide 1,2-distearoyl-snglycero-3- phosphocholine cholesterol.	Lipids (require lipids to help deliver the mRNA to the cells.) SM-102 1,2-dimyristoyl-rac-glycero3-methoxypolyethylene glycol-2000 (PEG2000-DMG) cholesterol 1,2-distearoyl-snglycero-3-phosphocholine (DSPC).	
Salts (to help balance the acidity in our body): potassium chloride, monobasic potassium phosphate, sodium chloride, dibasic sodium phosphate dihydrate.	Salt: sodium acetate	Salt: trisodium citrate dihydrate
Sugar (helps the molecules maintain their shape during freezing). Basic table sugar, also known as sucrose, can also be found in the new COVID vaccine. This ingredient helps the molecules maintain their shape during freezing.	Sugar: sucrose	Sugars: 2-hydroxypropyl-β-cyclodextrin (HBCD) polysorbate-80, sodium chloride
	Acids: acetic acid, acid stabilizers, tromethamine and tromethamine hydrochloride	Acids: citric acid monohydrate
-	-	Other ingredients: ethanol

Myocarditis is not a novel complication after the vaccine. The majority of the cases of perimyocarditis reported in a prospective safety surveillance study of the smallpox vaccine were subclinical. The cases were identified using routine troponin level checks before and post-immunization, according to the investigators [29]. Systemic adverse events following the second dose were recorded more frequently in the clinical trials, primarily in the younger demographic, with a median onset time of one to two days. Myocarditis, on the other hand, might have a more severe clinical presentation, limit physical activity, and necessitate long-term medical treatment and follow-up [19].

Male predominance in myocarditis/pericarditis instances has previously been documented in clinical and experimental studies, with the reasons for this being unknown. Differences in sex hormones are one key likely explanation. Testosterone is assumed to play a role through a combination of anti-inflammatory cell inhibition and commitment to a Th1 immune response. Estrogen inhibits pro-inflammatory T cells, resulting in a reduction in cell-mediated immune responses and thus reducing the risk of autoreactivity [30]. This explains the majority of reported cases in males (90%) compared to females (10%) in this study.

Myocarditis has an atypical clinical appearance. Unusual dyspnea, palpitations, chest discomfort with or without elevated troponin, arrhythmia, syncope, acute or chronic congestive heart failure, aborted abrupt cardiac death, and fulminant cardiogenic shock are all red flags [31]. A majority of cases of myocarditis following COVID-19 vaccination were reported two to three days after receiving the second dose of mRNA vaccination; all of the patients in these reports complained of chest pain [17].‌ Our analysis results also showed that the majority of patients presented with complaints of chest pain or shortness of breath within an average of 5.09 days (±14.04 days) after the administration of the COVID-19 vaccine.

EKG and cardiac troponin levels should be acquired for early examination, and inflammatory indicators including C-reactive protein and erythrocyte sedimentation rate can be helpful [32]. Cardiology consultation and examination using echocardiography and cardiac MRI should be considered in suspected situations. It would be beneficial to check for acute COVID-19 infection (through a polymerase chain reaction of a respiratory tract sample) and prior disease (using SARS-CoV-2 nucleocapsid and spike protein antibodies) [30]. Cardiac MRI adds to the picture and is especially helpful when an echocardiogram is not conclusive. CMR tissue characterization with T1 and T2 weighted imaging and late gadolinium enhancement (LGE), as well as parametric mapping, can be used to identify myocarditis. Myocarditis has a distinct subepicardial or mid-myocardial LGE pattern that allows it to be distinguished from coronary artery disease (CAD) [31]. In this analysis, the majority of the cases described had LGE patterns and/or myocardial edema found on cardiac MRI.

The gold standard for suspected myocarditis is an endomyocardial biopsy (EMB), which distinguishes between infectious and non-infectious myocarditis. In published case reports, nonsteroidal anti-inflammatory medications, prednisolone, and colchicine were used to treat some of the individuals who developed myocarditis after receiving the COVID-19 vaccination, in addition to supportive care. Because of left ventricular systolic dysfunction, a few patients were given intravenous immunoglobulin and aspirin, while others were started on beta-blockers and angiotensin-converting enzyme inhibitor medication [30]. Given the established risk of COVID-19 infection sequelae, such as hospitalizations and death, even in younger adults (mortality remains 0.1-1 per 100,000 for those 12-29 years old), the risk-benefit choice for immunization remains overwhelmingly beneficial. As a result, everyone over the age of 12 should get vaccinated against COVID-19 [30]. The incidence, risk factors, including genetic predisposition, prognosis, probable mechanisms, reasons for sex variations, clinical course, treatment methods, and long-term consequences of myocarditis after COVID-19 vaccination all require further research [32].

Strengths and limitations

This is the first large epidemiological study where we reviewed 40 case reports of COVID-19 vaccine-related myocarditis. A few limitations of this study are that not all case reports mentioned vitals and comorbidities for each patient. Some of the case reports did not mention time to recovery and treatment used. Another major limitation of this study is the inability to determine the disease progression and associated metabolic comorbidities. As this is a retrospective study of 40 case reports, it has an inferior level of evidence compared with prospective studies. This study has limited generalizability because of the smaller sample size and is prone to confounding bias. It cannot determine causation but can only talk about the association. Another limitation is that we have not included non-FDA-approved vaccines other than Pfizer, Moderna, and Janssen in this study. Most of the rest of the world has been vaccinated by various other vaccines, and their data were not accounted for in this study.

## Conclusions

Although rare, clinicians should be aware of the possibility of myocarditis and pericarditis, which should be taken into account in patients who report chest pain within a week of vaccination, particularly in the younger population. Further research should be conducted to study more on the risk-benefit ratio of COVID-19 vaccines.
